# Promoting Life Skills and Preventing Tobacco Use among Low-Income Mumbai Youth: Effects of Salaam Bombay Foundation Intervention

**DOI:** 10.1371/journal.pone.0034982

**Published:** 2012-04-16

**Authors:** Glorian Sorensen, Prakash C. Gupta, Eve Nagler, Kasisomayajula Viswanath

**Affiliations:** 1 Dana-Farber Cancer Institute, Center for Community-Based Research, Boston, Massachusetts, United States of America; 2 Harvard School of Public Health, Boston, Massachusetts, United States of America; 3 Healis, Sekhsaria Institute for Public Health, Navi-Mumbai, India; Fundación para la Prevención y el Control de las Enfermedades Crónicas No Transmisibles en América Latina (FunPRECAL), Argentina

## Abstract

**Background:**

In response to India's growing tobacco epidemic, strategies are needed to decrease tobacco use among Indian youth, particularly among those who are economically disadvantaged. The objective of this study was to assess the effectiveness of a school-based life-skills tobacco control program for youth of low socio-economic status in Mumbai and the surrounding state of Maharashtra. We hypothesized that compared to youth in control schools, youth exposed to the program would have greater knowledge of effects of tobacco use; be more likely to take action to prevent others from using tobacco; demonstrate more positive life skills and attitudes; and be less likely to report tobacco use.

**Methods/Findings:**

Using a quasi-experimental design, we assessed program effectiveness by comparing 8^th^ and 9^th^ grade students in intervention schools to 8^th^ grade students in comparable schools that did not receive the program. Across all schools, 1851 students completed a survey that assessed core program components in early 2010. The program consisted of activities focused on building awareness about the hazards of tobacco, developing life skills, and advocacy development. The primary outcome measure was self-reported tobacco use in the last 30 days.

Findings indicate that 4.1% of 8^th^ grade intervention students (OR = 0.51) and 3.6% of 9^th^ grade intervention students (OR = 0.33) reported using tobacco at least once in the last 30 days, compared to 8.7% of students in the control schools. Intervention group students were also significantly more knowledgeable about tobacco and related legislation, reported more efforts to prevent tobacco use among others, and reported stronger life skills and self-efficacy than students in control schools. Limitations to the study include schools not being randomly assigned to condition and tobacco use being measured by self-report.

**Conclusions:**

This program represents an effective model of school-based tobacco use prevention that low-income schools in India and other low- and middle-income countries can replicate.

## Introduction

Tobacco use is expected to contribute to an increasing proportion of the global disease burden [1], [2]. The World Health Organization estimates that deaths due to tobacco use will increase to 8 million by 2030 with 80% of them coming from the developing world [3]. Redoubling of efforts to combat this significant public health threat is needed, and interventions to decrease tobacco use among youth are a critical component of these strategies. Efforts targeting youth require a multi-pronged approach, including effective school programs, increased excise taxes, media campaigns, and community interventions that decrease children's access to tobacco [2], [4], [5].

India represents an important setting for such efforts, as the second largest country in the world and with prevalent use of multiple forms of tobacco. In 2010, one million Indians were expected to die from tobacco-related causes [6]. According to the 2009 Global Youth Tobacco Survey, 15% of Indian school children aged 13–15 years consumed tobacco (19% boys and 8% girls) in some form, and use is increasing among urban youth [7], [8]. Furthermore, it is well-established that in many countries socioeconomic position is a major factor in tobacco use, with the uneducated and those with lower incomes having higher levels of consumption [9], [10], a trend that holds as well for Indian youth. A recent study of students in Delhi and Chennai showed those attending government schools, which serve students from lower socioeconomic status backgrounds, had a higher prevalence of tobacco use compared with students from higher socioeconomic status attending private schools (18.9% vs. 12.2%, respectively) [8]. These disparities underscore the need for strategies to address tobacco control particularly among economically-disadvantaged youth.

There is a strong evidence base for tobacco use prevention interventions in developed countries [11]. Synthesis of findings from school-based tobacco control programs has suggested that programs that use a social influences approach, including education about social norms and teaching skills to address social influences, are most likely to be effective [5], [11]. Similarly, emerging research within India [12] and other developing nations [13], [14] and in Europe [15] demonstrate promise for the potential impact of school-based programs. Accordingly, it is imperative that steps be taken to close the gap between evidence-based strategies tested in research studies, and their application in practice [16]–[20]. Such research cannot rely exclusively on randomized controlled trials [21]–[23], but must increasingly include pragmatic, practice-based research that evaluates existing programs developed in response to the needs of local communities. If found effective, these programs hold promise for being readily implemented in other “real-world” settings [24].

This paper describes such an evaluation. Specifically, this study evaluated a tobacco use prevention program already in place in India, implemented by the Salaam Bombay Foundation (SBF). The aim of this program is to reduce tobacco use initiation and prevalence among youth from low socio-economic backgrounds in Mumbai and more broadly, in the Indian state of Maharashtra. SBF is a non-profit organization established in 2002 with the vision of “empowering children to live their life free from the threat of tobacco and to become confident to lead tomorrow's India” [25]. The program uses a holistic approach focusing on life skills, based on the premise that tobacco uptake and consequent addiction are a result of low self-esteem, lack of refusal skills and the inability to deal with peer pressure, further exaggerated by difficult living conditions in their target group children.

The purpose of this paper is to describe the results of an assessment of the effectiveness of the SBF program by comparing knowledge, attitudes and life skills, and tobacco use patterns among 8^th^ and 9^th^ grade students in SBF schools with 8^th^ grade students in schools not receiving the SBF program. We hypothesized that compared to students in control schools, students in schools receiving the SBF program will be: (1) more knowledgeable about products containing tobacco and about tobacco control legislation; (2) more likely to take action to prevent others from using tobacco; (3) more likely to demonstrate positive life skills and attitudes; and (4) less likely to report using tobacco in the last 30 days. This important study has the advantage of examining a program already being implemented on a large scale, with demonstrated infrastructure already in place.

## Methods

### Study design

This study used a quasi-experimental design to assess the efficacy of the SBF program in improving students' life skills and self-efficacy, actions against tobacco, and tobacco use. SBF leadership requested an independent evaluation of its program from this investigator team. In early 2010, surveys were administered to 8^th^ grade and 9^th^ grade students in randomly selected classes in schools receiving SBF programs, and to 8^th^ grade students similarly selected in control schools not receiving the SBF program. We compared the two 8^th^ grade conditions in order to estimate the program's effect after one year of intervention, and compared the 8^th^ grade control schools and 9^th^ grade intervention schools to estimate the its effect after two years of exposure to the program, while controlling for age, gender, and mother's education as an indicator of socioeconomic status.

### Intervention

The mission of SBF is to guard the next generation from the harmful effects of tobacco by working with children from resource-poor schools that cater to low socio-economic status populations, to reduce tobacco use initiation, and to foster the development of life and advocacy skills among these students. Program activities were funded by income from corpus funds of SBF; 30 full-time staff members delivered the program to the 8^th^ and 9^th^ grades across all schools participating in the program. During the 2010–11 academic year, SBF provided in-school programming to 49,866 children aged 10–17 from 147 government-run schools in Mumbai. Since its inception in 2002, SBF has trained 453,221 children and 16,029 teachers from schools in 17 districts of Maharashtra state in India.

The SBF program is designed to assist children in making informed decisions using valid information and the relevant life-skill tools to deal with life's challenges. The immediate objectives of the program include to: reduce initiation of tobacco use among participating children; create awareness on the harmful health consequences of tobacco and of misleading tobacco advertisements; create awareness of tobacco control legislation; build advocacy skills; provide sports and arts platforms to inculcate the concept of team work; develop leadership skills and create positive role models among peers; and build confidence, decision making skills, refusal skills and communication skills in order to handle peer pressure to stay away from tobacco.

The SBF program targets and objectives extend across a continuum from building awareness about the hazards of tobacco use among students in all participating schools, to developing life skills through a range of experiential platforms. These efforts are promoted through in-school programs focused in the first year (for 8^th^ graders) on awareness building, and in the second year (for 9^th^ graders) on advocacy training. Additional after-school programs offer “academies” that use the vehicles of sports, arts and journalism to build confidence, peer relations, and refusal skills. During Year 1, the “super army” offered within the classroom focuses on creating awareness of tobacco, as well as personality development that focuses on improved communication, refusal skills, handling peer pressures, and habit formation. In Year 2, students are trained to work with different civic authorities to support the implementation of the prevailing tobacco control law. Students interact with the police, the media, and health departments, and use religious and cultural festivals to involve the communities.

All sessions are divided into classroom and out-of-classroom activities. The classroom-based sessions were conducted once or twice a month for all children, with the aim of providing each child a minimum exposure of 10 one-hour classroom sessions per year. Attendance was tracked, and a minimum of 70% attendance was required for any session to be conducted; in cases where attendance was below 70%, the session was postponed and offered at a later date. The out-of-classroom activities were conducted regularly, two to three times a week with a focus on creating peer leaders. In this paper, we examine the impact of the program for 8^th^ graders after one year of exposure to the program, and for 9^th^ graders exposed to two years of in-classroom programming.

### Sample

We conducted a survey of students in schools receiving the SBF intervention, including 8th grade students from 20 schools and 9th grade students from 16 different schools, and from 8th grade students in 23 similar schools not receiving the SBF program. All schools were municipally funded, to be distinguished from other types of schools relying on private funds; accordingly, the schools in both groups represent similar student populations from low-income communities. One classroom per school was randomly selected to participate in the survey. Each classroom included between 25 and 70 students; approximately 30 students per class were randomly selected to participate in the survey. All students invited to participate responded to the survey. Approximately 5% of 8^th^ grade students in both conditions and 14% of 9^th^ grade students were absent from class the day of the survey.

### Survey and data collection

We developed a structured questionnaire with 73 questions, without any skip or branch patterns. The survey and data collection methods were pre-tested in two SBF schools and two non-SBF schools that were not part of the study. The survey was translated and administered in Hindi and Marathi, depending on the language of instruction within the school. The questions and instructions were read aloud to the class and responses were marked by each student on the questionnaire. This survey administration method was preferred over a fully self-administered format given the literacy levels of the students. Data were collected in February 2010, which marks the end of the academic calendar in these schools. The survey administrators were trained in standard data collection methods, and were independent of the SBF staff. The survey was completely anonymous.

The survey was designed to assess primary and secondary outcomes of participation in the SBF program, as outlined in the hypotheses. To the extent possible, items were adapted from existing instruments [26], [27]. The survey assessed the primary outcome, current tobacco use, based on responses to the question, “In last month, how many days did you use Gutkha, Mava, Mishri, Khaini, Pan Masala, Cigarette or Bidi?” Respondents using tobacco on one day or more were considered current users. We measured secondary outcomes related to tobacco use, specifically, knowledge of products containing tobacco and of tobacco control legislation (see items listed in Table 1) and actions taken to prevent others from using tobacco (see items listed in Table 2) . We additionally measured secondary outcomes related to life skills and attitudes, including self-efficacy and confidence about one's life prospects (see items listed in Table 3).We also assessed exposure to SBF activities, focusing on awareness of SBF activities and having read the SBF newsletter. Sociodemographic characteristics measured included age and gender; education level of the student's mother was used to indicate socio-economic status.

**Table 1:** Knowledge of tobacco and tobacco control legislation: Comparisons of responses from 8^th^ grade control schools, 8^th^ grade intervention schools and 9^th^ grade intervention schools.

	Agree/Yes N (%)			8^th^ grade control vs. 8^th^ grade Intervention		8^th^ grade control vs 9^th^ grade Intervention	
	8^th^ Grade Control	8^th^ Grade Intervention	9^th^ Grade Intervention	Adjusted Odds Ratio	95% CI	Adj Odds Ratio	95% CI
Does gutkha contain tobacco?(%yes)	194 (28.2)	292 (44.2)	224(44.7)	2.2	1.7–2.8	2.1	1.6–2.8
Does mishri contain tobacco? (%yes)	379 (55.1)	403 (61.0)	368(73.5)	1.3	1.0–1.6	1.9	1.4–2.5
Which product is found in gutkha, mava, mishri, khaini, pan masala, cigarette, bidi?*	226 (32.9)	497 (77.3)	417(83.9)	7.4	5.7–9.6	22.8	15.6–33.4
Is there a law in Mumbai which stops people from smoking in public places? (%yes)	374 (54.3)	414 (62.6)	347(69.3)	1.4	1.1–1.8	2.0	1.5–2.7
In Mumbai is it against the law to sell tobacco to minors under age 18? (%yes)	437 (63.5)	465 (70.3)	431(86.0)	1.5	1.1–1.9	3.5	2.5–5.0

* Response 1: Nicotine; Response 2: Others.

Odds ratios adjusted for age, gender, mother's education and school.

**Table 2:** Actions taken to prevent tobacco use: Comparisons of responses from 8^th^ grade control schools, 8^th^ grade intervention schools and 9^th^ grade intervention schools.

	Agree/yes N (%)			8th Grade Control vs 8th Grade Intervention		8th Grade Control vs. 9th Grade Intervention	
	8th Grade Control	8th Grade Intervention	9th Grade Intervention	Adj Odds Ratio	95% CI	Adj Odds Ratio	95% CI
Do you think you could help a friend to stay away from trying gutkha, mava, etc.?	529 (77.0)	554 (83.8)	437(87.2)	1.5	1.1–2.0	2.5	1.7–3.6
In last year, have you worked to prevent or reduce tobacco use in your neighbourhood?	124 (18.1)	148 (22.4)	106(21.2)	1.4	1.1–1.9	1.1	0.8–1.5
In last year, have you worked to prevent or reduce tobacco use in your school?	217 (31.5)	356 (53.9)	360(71.9)	2.8	2.2–3.5	4.6	3.4–6.1
It is none of my business to tell other people not to use tobacco.	351 (51.1)	393 (59.5)	300(60.3)	1.5	1.2–1.8	1.5	1.1–2.0

Odds ratios adjusted for age, gender, mother's education and school

**Table 3:** Attitudes and life skills: Comparisons of responses from 8^th^ grade control schools, 8^th^ grade intervention schools and 9^th^ grade intervention schools.

	Agree/Yes N (%)			8th Grade Control vs. 8th Grade Intervention		8th Grade Control vs. 9th Grade Intervention	
	8th Grade Control	8th Grade Intervention	9th Grade Intervention	Adj Odds Ratio	95% CI	Adj Odds Ratio	95% CI
I believe my life has no purpose	40′ (58.2)	257 (38.9)	184(36.7)	0.45	0.4–0.6	0.40	0.3–0.5
Nobody cares what my opinion is	435 (63.3)	341 (51.7)	274(54.8)	0.66	0.5–0.8	0.65	0.5–0.9
I don't know if I will study till 10^th^ (grade).	565 (82.0)	421 (63.8)	349(69.8)	0.40	0.3–0.5	0.49	0.4–0.7
I worry about my ability to support myself financially in the future	591 (86.0)	467 (70.8)	349(69.6)	0.38	0.3–0.5	0.32	0.2–0.5
I don't know what will happen to me after I finish 10^th^ (grade)*	399 (58.0)	422 (63.8)	302(60.3)	0.81	0.6–1.0	0.83	0.6–1.1
I can face the world with confidence	625 (90.7)	618 (93.8)	475(95.0)	0.64	0.4–1.0	0.54	0.3–1.0
I can travel alone by train/bus anywhere in Mumbai	499 (72.4)	500 (76.0)	421(84.1)	0.74	0.6–1.0	0.63	0.4–0.9

* Response 1: I don't know; Response 2: I know.

Odds ratios adjusted for age, gender, mother's education and school.

### Statistical Analyses

The statistical analysis was conducted using a random effects logistic regression model adjusted for age (< = 14 yr, > = 15 yr), gender, mother's education and clustering of respondents within school.

### Ethics

This research was approved by the Institutional Review Board of the Healis – Sekhsaria Institute for Public Health. Standard procedures for protection of human subjects were used, and were similar to those used in collection of data for the Global Youth Tobacco Surveys. Written permission was obtained from the Education Department of the Municipal Corporation that operates all those schools; SBF facilitated entry of the survey staff in the school. Verbal informed consent from school authorities was obtained. Informed consent was not sought from parents/guardians of children; this was not feasible given schools' minimal contact with low-income parents with low literacy. Careful steps were taken to assure ethical safeguards were in place, including explaining the voluntary nature of participation in the survey; keeping the answer sheets completely anonymous, ensuring no participation from class teachers or school authorities during the administration of the survey; collecting only the relevant information concerning tobacco use and no other sensitive information of any kind; and commitment to not disclosing partial results to schools.

## Results

### Sample characteristics

The 1851 respondents to the survey included 690 from the 23 8^th^ grade control schools (total number enrolled students = 1054), 660 from the 20 8^th^ grade intervention schools (total number enrolled students = 1045), and 501 from the 16 9^th^ grade intervention schools (total number enrolled students = 800).

The intervention and control conditions were fairly well balanced by gender. Among 8^th^ grade students, there were49% (n = 340) girls in the control group vs. 52% (n = 341) in the intervention group, although there were somewhat more girls in the 9^th^ grade intervention classes (54%, n = 272). Among 8^th^ graders, the intervention group included a somewhat larger proportion of students 14 years of age and under relative to the control group (81.5%, n = 539, compared to 73.2%, n = 503). The average age of 8^th^ grade control group was 13.6 yrs, of 8^th^ grade intervention group, 13.4 yrs, and of 9^th^ grade intervention group, 14.5 yrs. We also compared the groups by mother's education as an indicator of socioeconomic status; 21% (n = 135) of mothers of 8^th^ grade control group students received education at the 8^th^ standard and above, compared with 28% (n = 174) of mothers in 8^th^ grade intervention group and 37% (n = 187) of mothers in 9^th^ grade intervention group. The differences in mothers' education between intervention and control groups were significant (p<.01); further analyses control for mother's education.

### Exposure to SBF activities

To assess awareness of SBF activities, we asked students in both conditions if they had heard of SBF; 16% of 8^th^ graders in the control group responded “yes,” compared with 97% of 8^th^ graders in intervention schools and 99% of 9^th^ graders in intervention schools. We also asked respondents if they read “Halla Bol,” the SBF newsletter; 5% of 8^th^ graders in the control group responded “yes,” compared with 50% of 8^th^ graders in intervention schools, and 40% of 9^th^ graders in intervention schools. The between-group differences were statistically significant (p<0.01).

### Differences in knowledge

As shown in Table 1, we observed significant between-group differences in knowledge about tobacco and tobacco control legislation, adjusted for age, gender, school and mother's education. Compared to 8^th^ grade students in control schools, intervention students were significantly more likely to report the presence of tobacco in gukta and mishri. In addition, intervention students in both grades were significantly more likely to know that nicotine was the common ingredient in a range of tobacco products. Students in intervention schools were also significantly more knowledgeable about tobacco control legislation compared to 8^th^ grade students in control schools, including about a law prohibiting people from smoking in public places, and a law against selling tobacco products to minors.

### Differences in actions taken to prevent tobacco use

Responses to the survey indicate that students in intervention schools were significantly more likely to take key actions to prevent tobacco use in the communities and among their friends, adjusted for age, gender, school, and mother's education, as illustrated in Table 2. Responses also indicate that 8^th^ grade intervention students were more likely than control group 8^th^ graders to report that they had “worked to prevent or reduce tobacco use in (their) neighborhood”, although these between-group differences were not significant between 8^th^ grade control and 9^th^ grade intervention schools. SBF students were significantly more likely to believe they could prevent a friend from using tobacco.

### Differences in attitudes and life skills

As noted in description of the intervention, SBF aimed to build life skills as a core strategy in tobacco use prevention. Indicators of life skills and self-efficacy were higher among students in SBF schools. As presented in Table 3, adjusted for age, gender, school, and mother's education, compared to control school students, those in intervention schools were less likely to agree that “my life has no purpose”; “I don't know if I will study till 10th”; “I worry about my ability to support myself financially in the future”; and “nobody cares what my opinion is”.

### Differences in self-reported tobacco use

Students in SBF schools were significantly less likely to report using some form of tobacco. Among 8^th^ grade control school students, 8.7% reported using tobacco for at least one of the last 30 days, compared to 4.1% of 8^th^ grade intervention students (OR = .0.51, 95% CI = 0.3–0.8) and 3.6% of 9^th^ graders in intervention schools (OR = 0.33, 95% CI = 0.2–0.6), adjusted for age, gender, school, and mother's education.

## Discussion

Established in 2002, the SBF program was designed to reduce tobacco use initiation among children from low socio-economic backgrounds attending government schools in Mumbai, India, following an innovative approach focused on building broad-based life skills and confidence to address life's challenges. This study used a quasi-experimental design to test the effectiveness of this program by comparing tobacco use and related knowledge and attitudes among students in SBF schools and in comparable control schools in Mumbai that have not received the SBF intervention. Specifically, we tested the hypotheses that compared to students in control schools, students in schools receiving the SBF program would be more knowledgeable about products containing tobacco and about tobacco control legislation; more likely to take action to prevent others from using tobacco; more likely to demonstrate positive life skills and attitudes; and less likely to report using tobacco in the last 30 days. Notably, these findings indicate that even after only one year of exposure to the program, students in SBF schools were only half as likely as students in control schools to have used tobacco in the last 30 days, and the proportion using tobacco after two years of exposure to the program was even further reduced. Reflecting the central premise of the SBF program, SBF students also reported stronger life skills and self-efficacy than students in control schools. In addition, compared to control school students, SBF students were significantly more knowledgeable about tobacco and related legislation, and reported significantly more efforts to prevent tobacco use among others, including with friends, in their schools and in their neighborhoods.

These findings are consistent with prior studies, including the emerging literature on tobacco use prevention efforts in India. For example, our findings about SBF students' improved knowledge about products containing tobacco and about tobacco control legislation are consistent with a study of students in New Delhi. This study found greater knowledge of tobacco, including types of tobacco and its harmful effects, was associated with lower levels of tobacco use compared to students with less knowledge of tobacco [28]. India's Cigarettes and Other Tobacco Products Act (COTPA) of 2003 is relatively new and not well enforced in many parts of the country [29]. The 9^th^ grade SBF intervention included students working with civic authorities to support implementation of the tobacco control law; knowledge of this legislation provided an important foundation for their advocacy work.

We also found support for our hypothesis that SBF students would be more likely to take action to prevent others from using tobacco than control school students. Other studies have noted a crucial role for peer activism and engagement in successful programs designed to prevent youth tobacco use [30]–[33]. SBF engaged youth in multiple ways and at multiple levels in tobacco use prevention efforts, including at the individual level (friends), the organizational level (schools) and community level (neighborhoods). The use of multiple modalities and domains to deliver program content has been shown to be more effective in reducing tobacco use than in-school activities only [5], [31], [34], [35].

Students in the SBF schools were more likely to demonstrate positive life skills and attitudes than students in the comparison schools. A wealth of research over the past two decades has demonstrated both short- and long-term prevention effects of life skills training on tobacco use [31]. These effects have been found in a variety of school settings and student populations [5], [36]–[38]. Given that low self-esteem has been found to be an independent predictor of smoking initiation in adolescents [39], SBF's life skills training may confer future tobacco prevention benefits, beyond the duration of the program.

We are aware of only one other school-based intervention study to reduce tobacco use among adolescents in India, based in Delhi and Chennai. Investigators found overall tobacco use increased by 68% in the control group and decreased by 17% in the intervention group over 2 years [12]. In this study, we found that 8^th^ grade students exposed to the SBF intervention for only one academic year were half as likely as students in comparison schools to have used tobacco in the last 30 days. Of particular note, we found that 9^th^ graders in SBF schools were even less likely to use tobacco (OR = 0.34). Outside the context of the SBF program, one would expect to see tobacco use uptake rates increase from 8^th^ grade to 9^th^ grade. This apparent decrease in tobacco use prevalence over time, reflected by the difference between 8^th^ and 9^th^ grade students exposed to the SBF intervention, suggests an increased dose effect with greater exposure to the intervention.

The life skills approach used in the SBF program differs in substantial ways from interventions focusing solely on tobacco education and may be particularly relevant for students from low-income communities, who often have restricted access for opportunities to build such life skills. A life skills approach is designed to enhance general personal and social competence, along with providing information and skills specific to tobacco use [40]. The goal of enhancing personal and social skills, such as decision-making skills, is to improve self-esteem, decrease motivations to use tobacco, and provide the necessary coping skills to manage social pressures to use. Providing information and skills specific to tobacco use helps promote resistance skills and fosters anti-tobacco attitudes and norms. This broad array of self-management and social skills has application beyond tobacco and can be used by youth in a variety of other settings.

Overall, these findings are consistent with prior reports. In a systematic review of school-based smoking prevention programs, Flay concluded that programs with 15 or more sessions (beginning in upper elementary/middle school and continuing to high school); were based on a social influences model; focused on changing social norms, commitments not to use and intention not to smoke; taught refusal and other life skills and engaged peers in the program delivery could reduce smoking onset by 25–50% [31]. Similarly, the Institute of Medicine concluded that school-based prevention programs that are interactive, teach about social influences, provide opportunities to practice social skills, result in a 12 percent reduction in the rate of initiation [5]. The SBF contains these elements.

It is important to note several caveats in the interpretation of these results. Tobacco use was measured by self report; even though data were collected by independent evaluators with no connection to the intervention, it is possible that findings could be influenced by a social desirability bias in the intervention schools. We used a quasi-experimental design, wherein schools were not randomly assigned to condition. Although control schools were specifically selected from other government-run schools in the same general locale, we acknowledge that some unmeasured differences between the schools (e.g., location, size) and between the students (e.g., migrant status) may contribute to finding differences between SBF and comparison schools. Nonetheless, we have controlled for age, gender, and mother's education in the analyses to account for potential between-group differences. In addition, it is important to acknowledge that these results can only be generalized to similar school settings. Comparison of 9^th^ grade intervention with 8^th^ standard controls has a limitation of difference in the years of schooling that could not be adjusted. The age distribution was also different but that has been adjusted for. In addition, it was not possible to evaluate the potential differential effects of individual components on the intervention, or to examine the long-term effects of this intervention on tobacco use prevention.

The strengths of this evaluation include its systematic approach to surveying students from both intervention and control schools, carefully controlling for between-group differences, and assuring independence between intervention and evaluation teams. Based on prior research and on-the-ground understanding of local priorities and practices, SBF created this innovative program and had been implementing it in government schools since 2002, before requesting this independent evaluation of the program's impact. Rigorous program evaluations of existing programs, such as this one, can help highlight effective strategies from the field and facilitate their dissemination to different settings.

In India, every day more than 5,500 children under age 15 try tobacco for the first time. Currently, an estimated 5 million Indian children are addicted to tobacco [41], [42]. Easy availability of tobacco and lack of social sanctions have made tobacco use a problem of epidemic proportions among children, especially in the lower economic strata. The combined strategies of building students' life skills, providing opportunities for advocacy efforts, changing social norms, and engaging students in their broader communities are central to this tobacco use prevention program. By working closely with the government educational structures to embed these interventions in local infrastructures, SBF has increased the reach of the program and established it as part of institutional practice. This study was among the first life-skills intervention for tobacco use prevention to demonstrate an impact on youth tobacco use in India. This intervention additionally resulted in improved perceptions of one's life prospects and self-efficacy, and increased actions taken to prevent tobacco use among others. Accordingly, the SBF program represents an effective model of school-based tobacco use prevention that low-income schools in India and other low- and middle-income countries can replicate.
